# Protein subcellular localization and functional studies in horticultural research: problems, solutions, and new approaches

**DOI:** 10.1093/hr/uhac271

**Published:** 2022-12-02

**Authors:** Ye Guo, Zhiru Bao, Yaling Deng, Yuhui Li, Pengwei Wang

**Affiliations:** Key Laboratory of Horticultural Plant Biology (MOE), College of Horticulture and Forestry Sciences, Huazhong Agricultural University, Wuhan, Hubei Province 430070, China; Hubei Hongshan Laboratory, Wuhan 430070, China; Key Laboratory of Horticultural Plant Biology (MOE), College of Horticulture and Forestry Sciences, Huazhong Agricultural University, Wuhan, Hubei Province 430070, China; Hubei Hongshan Laboratory, Wuhan 430070, China; Key Laboratory of Horticultural Plant Biology (MOE), College of Horticulture and Forestry Sciences, Huazhong Agricultural University, Wuhan, Hubei Province 430070, China; Hubei Hongshan Laboratory, Wuhan 430070, China; Key Laboratory of Horticultural Plant Biology (MOE), College of Horticulture and Forestry Sciences, Huazhong Agricultural University, Wuhan, Hubei Province 430070, China; Hubei Hongshan Laboratory, Wuhan 430070, China; Key Laboratory of Horticultural Plant Biology (MOE), College of Horticulture and Forestry Sciences, Huazhong Agricultural University, Wuhan, Hubei Province 430070, China; Hubei Hongshan Laboratory, Wuhan 430070, China

To ensure different biochemical reactions take place simultaneously, eukaryotic cells have evolved several membrane-bounded organelles, which coordinate their functions through proteins involved in signal transduction, vesicle trafficking, and membrane interactions [1]. Therefore, determining the subcellular localization of a protein is an essential step toward understanding its functions and activities. To do this, people normally co-express genes of interest with a fluorescent organelle marker using the *N. Benthamiana* (*Nicotiana benthamiana*) transient expression system and image it with confocal microscopy[2]. This method is commonly used in horticulture research, where generating stable transgenic plants is still challenging for most species. Although there are a few common standards for this operation, such as signals should not be over-saturated, controls should be performed to eliminate the possibility of signal cross-talk between multiple channels (bleed-through), and the microscope settings (e.g. laser output, pinhole) should be kept identical when comparing two independent experiments, things may still go wrong in some circumstances. Here, we have highlighted a few examples of inappropriate image acquisition that happened in various previous studies and provide our reasonings and solutions to these issues.

Despite the potential issues of using a heterogeneous expression system, images taken from inappropriate focal planes may produce further difficulties to interpret the correct protein localization. For example, in mature leaf cells, more than 90% of the volume is occupied by the vacuole, and all cytoplasmic localized organelles are pushed towards the cell cortical region (Fig. 1a) [3]. The distance between fluorescent protein-labelled structures within this confined space is close to the resolution limit of normal light microscopy. Therefore, it is not possible to distinguish co-localizing signals from the background if images are taken from the middle section of a cell. As shown in Fig. 1b, free GFP or RFP localizes to the cytoplasm, and their signals are distinct from most subcellular structures (e.g cytoskeleton, mitochondria). However, if images were taken from the middle section, it is then impossible to tell the differences, and the signal distribution analysis tends to indicate strong co-localizations in all tests (Fig. 1c and d). This is problematic when studying proteins associated with cytoplasmic structures or localized to the cytosol, as their localization may become indistinguishable from the plasma membrane (PM) or cytosolic background. To solve this problem and achieve the best resolution of most organelles (except for the nucleus), we recommend that images should be taken from the cortical section of leaf epidermal cells, which is the cytoplasmic layer (approximately 1 μm thick) that is positioned in-between the vacuole membrane (tonoplast) and the plasma membrane (Fig. 1a).

**Figure 1:**
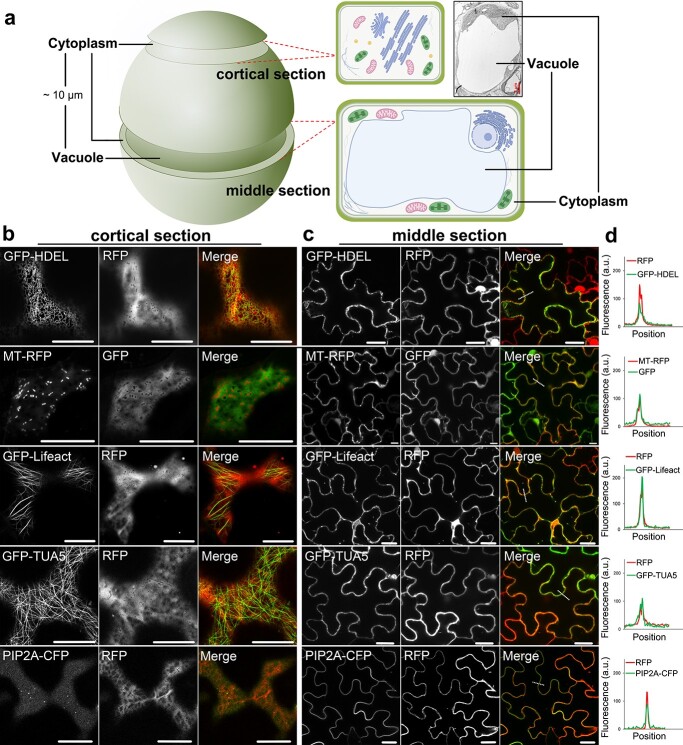
Transient expression of FPs-tagged organelle markers in *N. benthamiana* leaf epidermal cells. **a** Illustrations of images taken from different Z axial positions. In the middle section, the central vacuole occupied most of the space and the cytoplasm is hard to detect in most areas of the cell. **b** Subcellular localization of various organelles and cytoskeleton markers in the cortical region of *N. benthamiana* leaf epidermal cells. GFP-HDEL, endoplasmic reticulum; MT-RFP, mitochondria; GFP-Lifeact, actin cytoskeleton; RFP-TUA5, microtubules; PIP2A-CFP, plasma membrane. Free GFP or RFP was co-expressed as a reference of cytoplasm. **c** The same FP markers as used in panel **b**, but all images were taken from the middle section. **d** The distributions and intensities analysis of GFP and RFP signal along the dotted line indicated in **c**. a.u., arbitrary units. Scale bar = 10 μm for confocal; scale bar = 2 μm for TEM.

Furthermore, over-expressing some membrane proteins destined for secretion (e.g. to the PM or vacuole) may get trapped in some intermediate compartment and produce false labelling. For example, PIP2A (plasma membrane intrinsic protein 2A) is an aquaporin protein that is commonly used as a PM marker [2]. However, when its expression level is high, PIP2A accumulates in the ER and cannot be transported to the PM effectively (Fig. 2a). A similar effect is also likely found with other secretory membrane proteins at are produced in the ER. Therefore, it is critical to make sure that the marker proteins are localized correctly at the start of an experiment, and using a lower optical density (OD_600_ < 0.05) of *Agrobacterium* for infiltration may help (Fig. 2a). However, protein expression level varies from cell to cell, so it could be difficult for non-experienced researchers to determine cells with optimized protein expression and correct localization. To better overcome these problems, we have generated a collection of stable *N. Benthamiana* lines expressing FPs-tagged organelle marker [2]. These include the mitochondria marker, MT-RFP; the autophagosomes marker, GFP-ATG8a; the plasma membrane marker, PIP2A-CFP; the plastid marker, PT-RFP; the actin cytoskeleton marker, GFP-Lifeact; and the microtubule marker, RFP-TUA5. Fluorescent markers in these plants exhibit uniformed expression levels and correct subcellular localizations (Fig. 2b); therefore, they can be used for protein co-localization studies, producing more consistent results.

**Figure 2:**
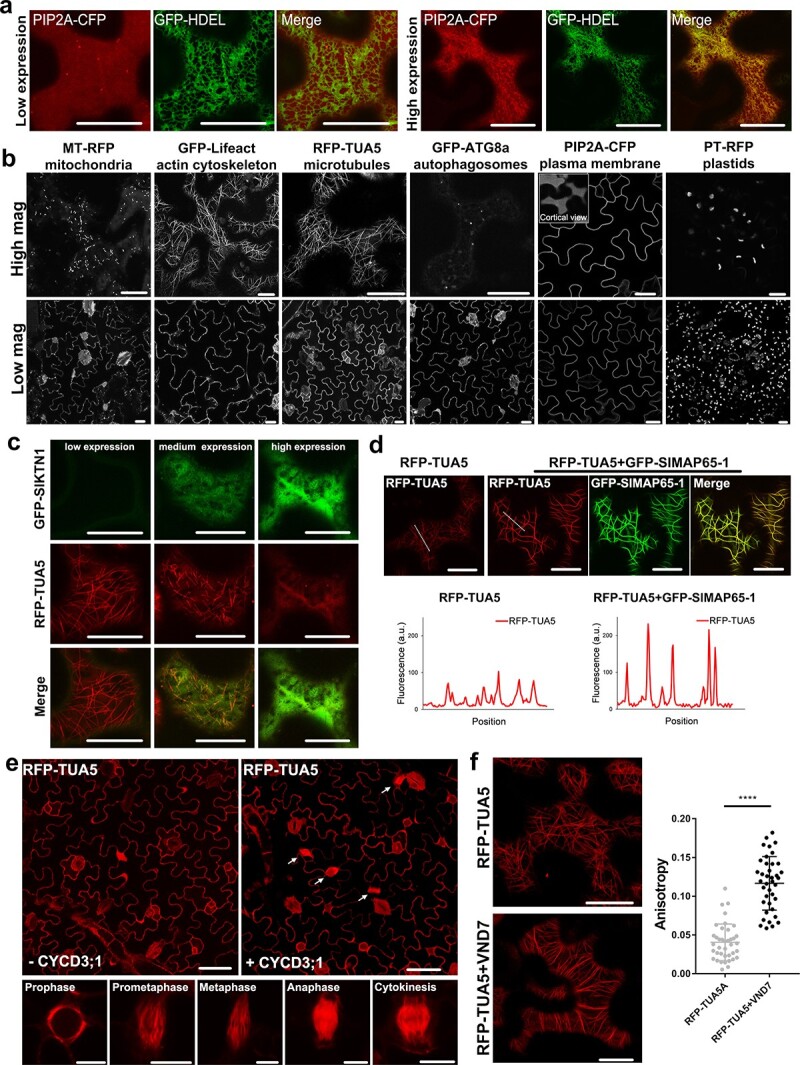
The application of stable *N. benthamiana* marker lines. **a** PIP2A-CFP is used as a plasma membrane marker at the optimized expression level, but it may also accumulate in the endoplasmic reticulum (labelled by GFP-HDEL) when its expression is high. **b** Subcellular localization of FPs-tagged organelle markers (mitochondria, actin cytoskeleton, cortical microtubules, autophagosome, plasma membrane, and plastids) in epidermal cells of stable transgenic tobacco. The top panel, images with high magnifications; the lower panel, images with low magnifications. **c** Different expression levels of GFP-SlKNT1 in transgenic *N. benthamiana* plants expressing the microtubule marker, RFP-TUA5. **d** SlMAP65-1 was expressed in the epidermal cells of the RFP-TUA5 line. Fluorescent intensities analysis (along the dotted line) suggested that the RFP signal of each filament is stronger in the presence of SlMAP65-1, indicating a microtubule bundling effect. **e** Transient expression of CYCD3;1 triggers tobacco epidermal cells to divide. White arrows point to cells in various stages of cytokinesis. The scale bars in the top row = 50 μm, and the scale bars in the bottom row = 10 μm. **f.** Transient expression of VND7 in RFP-TUA5 plants induces the re-organization and bundling of cortical microtubules. The changes in microtubule arrangement were quantified as the value of anisotropy. Values close to 0 indicate less ordered (isotropic arrays), while values close to 1 indicate perfectly ordered (parallel arrays). Asterisks denote significant differences relative to control as determined by Student's *t* test (^****^P < 0.0001). Scale bar = 10 μm, except for **e**.

Additionally, these stable *N. benthamiana* plants can also be used to characterize protein function at the subcellular level. Here, we used the RFP-TUA5 lines to study the functions of different microtubule regulators as examples. It is known that microtubules play an important role in plant development and fruit shape formation [4]. Therefore, we cloned the katanin p60 catalytic subunit (SlKTN1) and SlMAP65-1 of tomato (*Solanum lycopersicum*) and studied their localization and roles in cortical microtubule rearrangement. Transient transformed GFP-SlKTN1 fusion was found in the cytoplasm (Fig. 2c). However, with the increase of SlKTN1 expression, the degree of microtubule depolymerization is also enhanced, confirming the SlKTN1 from tomato has a function to cut microtubules, as reported in *Arabidopsis thaliana* [5]. Similarly, the transgenic plants were infiltrated with a 35S:GFP-SlMAP65-1, the protein not only co-localized with RFP-TUA5 labelled microtubules (Fig. 2d), but also induced microtubule bundles formation, suggesting the SlMAP65-1 from tomato may have the capacity to cross-link microtubules [6]. Because stable microtubule marker plants were used in the study, it is confident to say that the effects of SlMAP65-1 or SlKTN1 on microtubule structure were caused by the function of the proteins, not the variation of microtubule markers that were expressed at a different level. In the same way, other transgenic marker lines can be used to study the localization and activity of a protein in different subcellular compartments.

These transgenic lines can also be used to study the effect of transcription factors on subcellular dynamics. For example, CYCD3;1 is a D-type cyclin that increases mitotic cycles and reduces endocycles [7, 8]. When it was transiently transformed into the RFP-TUA5 line, characteristic microtubule arrays that represent phragmoplast or mitotic spindles were observed, suggesting the heterologous expression of CYCD3;1 in leaf epidermal cells is able to promote mitosis (Fig. 2e). The NAC domain transcription factor, VASCULAR-RELATED NAC-DOMAIN7 (VND7), is a key regulator of xylem vessel differentiation and secondary wall patterning [9]. When VND7 was transiently expressed in *N. benthamiana* leaf, it mimics the effect of xylem differentiation, where microtubules reorganized into thick bundles, which may act as the track to direct the movement of cellulose synthase complex and the deposition of cellulose microfibers (Fig. 2f). Similarly, other key transcription factors can be transiently transformed in these stable plants, and their functions from a cell biological perspective could be determined.

Taken together, determining the localization of a protein is the step forward in understanding the function of any particular molecular pathway. While the most promising results come from studies using the native species at the endogenous expression level, the transient expression system certainly provides a good alternative to rapid screening, especially useful for horticultural crops that are normally difficult for genetic transformation. Here, we have highlighted some common issues for bio-imaging and generated several stable transgenic *N. benthamiana* lines that can be used for studying protein localization and functions for further research.

## Materials and methods

### Transient and stable transformation of *N. benthamiana*

*Agrobacterium*-mediated transient expression of *N. benthamiana* was performed as described before [10, 11]. For stable transformation, surface-sterilized seeds of *N. benthamiana* plants were grown on Murashige and Skoog (MS) agar in a growth chamber with a 16 h : 8 h, light : dark regime under 25 degrees. Leaf segments of 4-week-old plants were inoculated with the *Agrobacterium* culture and co-cultivated for 2 days on MS media, supplemented with 6-Benzylaminopurine (6-BA; 1 mg l^−1^) and α-naphthalene acetic acid (NAA; 3 mg l^−1^). Putative transgenic shoots from the leaf explants were induced on the same medium supplemented with Carbenicillin salt (150 mg l^−1^) and Kanamycin Sulfate (50 mg l^−1^). Regenerated shoots were transferred to rooting media, including MS, with Carbenicillin salt (150 mg l^−1^). After rooting, the plants were transferred to soil and kept at 25°C with a 16-h photoperiod.

### Plasmid construction

The fluorescent markers GFP-HDEL, MT-RFP, PT-RFP, PIP2A-CFP, and GFP-Lifeact were described in the previous studies [2, 12]. The vector of 35S:AtCYCD3;1 was described by Xu *et al.*, 2020 [8]. The cDNAs of AtATG8a, SlHY5, AtTUA5, SlMAP65–1, SlKTN1, and AtVND7 were amplified from a seedling cDNA library with gene-specific primers (Table S1, see online supplementary material). The fluorescent fusion proteins were cloned into destination vectors pK7WGR2 (N-terminal RFP) or pMDC43-GFP (N-terminal GFP) using the Gateway cloning system (Invitrogen, California, UA).

### Image acquisition and analysis

Images were taken using a laser scanning confocal microscope (Leica TCS SP8) in multi-track mode with line switching, and processed with Fiji-ImageJ (https://imagej.net/imagej-wiki-static/Fiji). The signal distribution analysis of FPs-tagged organelle markers was performed using the Plot Profile tool. The FibrilTool plugin [13] was used to measure the overall anisotropy of microtubules in leaf epidermal cells (at least 30 cells were analysed) as described before [14].

## Acknowledgements

This work was supported by the National Key Research and Development Program (2019YFD1000103), NSFC grants (Nos 31772281, 92254307, 91854102), Foundation of Hubei Hongshan Laboratory (2021hszd016) to P.W., and Hubei province grant (2021CFB142) to Z.B.

## Data availability

The data used to support the findings of this study are available from the corresponding author upon request.

## Conflict of interest

The authors declare that they have no conflicts of interest.

## Supplementary data

Supplementary data is available at *Horticulture Research* online.

## Supplementary Material

Web_Material_uhac271
