# Supplementary material for: Protein subcellular localization and functional studies in horticultural research: problems, solutions, and new approaches
Source: Hortic Res. 2022 Dec 2;10(2):uhac271. doi: 10.1093/hr/uhac271 (PMC9923208; doi:10.1093/hr/uhac271)
Supplement: Web_Material_uhac271 [file web_material_uhac271.docx]

Table S1. Primers used in this study

| AtTUA5-F | AAAAAGCAGGCTCCATGAGGGAAATTATTAG |
| --- | --- |
| AtTUA5-R | AGAAAGCTGGGTGTCAATAGTCTTCACCTTC |
| SlMAP65-1F | AAAAAGCAGGCTCCATGGCAGCAGTAGATGATCA |
| SlMAP65-1R | AGAAAGCTGGGTGCTATGGGGTGCTAGGAATAG |
| SlKTN1-F | AAAAAGCAGGCTCCATGGTGGGTGCATCACTGG |
| SlKTN1-R | AGAAAGCTGGGTGCTAAGCTGATCCAAATTCTG |
| SlHY5-F | AAAAAGCAGGCTCCATGCAAGAGCAAGCGACGAG |
| SlHY5-R | AGAAAGCTGGGTGCTTCCTCCCTTCCTGTGCACC |
| AtATG8a-F | AAAAAGCAGGCTCCATGATCTTTGCTTGCTTGAAATTCG |
| AtATG8a-R | AGAAAGCTGGGTGTCAAGCAACGGTAAGAGATCCAA |
| AtVND7-F | AAAAAGCAGGCTCCATGGATAATATAATGCAATCGTCAATGC |
| AtVND7-R | AGAAAGCTGGGTGTTACGAGTCAGGGAAGCATCCAA |
